# Seasonality of inundation in geographically isolated wetlands across the United States

**DOI:** 10.1088/1748-9326/ac6149

**Published:** 2022-04-19

**Authors:** Junehyeong Park, Mukesh Kumar, Charles R Lane, Nandita B Basu

**Affiliations:** 1 Department of Civil, Construction and Environmental Engineering, University of Alabama, Tuscaloosa, AL, United States of America; 2 US Environmental Protection Agency, Office of Research and Development, Cincinnati, OH, United States of America; 3 Department of Civil and Environmental Engineering, University of Waterloo, Waterloo, ON, Canada

**Keywords:** GIW, remote sensing of wetlands, non-floodplain wetlands, wetland dynamics, wetland area

## Abstract

Inundation area is a major control on the ecosystem services provisioned by geographically isolated wetlands. Despite its importance, there has not been any comprehensive study to map out the seasonal inundation characteristics of geographically isolated wetlands over the continental United States (CONUS). This study fills the aforementioned gap by evaluating the seasonality or the long-term intra-annual variations of wetland inundation in ten wetlandscapes across the CONUS. We also assess the consistency of these intra-annual variations. Finally, we evaluate the extent to which the seasonality can be explained based on widely available hydrologic fluxes. Our findings highlight significant intra-annual variations of inundation within most wetlandscapes, with a standard deviation of the long-term averaged monthly inundation area ranging from 15% to 151% of its mean across the wetlandscapes. Stark differences in inundation seasonality are observed between snow-affected vs. rain-fed wetlandscapes. The former usually shows the maximum monthly inundation in April following spring snowmelt (SM), while the latter experiences the maximum in February. Although the magnitude of inundation fraction has changed over time in several wetlandscapes, the seasonality of these wetlands shows remarkable constancy. Overall, commonly available regional hydrologic fluxes (e.g. rainfall, SM, and evapotranspiration) are found to be able to explain the inundation seasonality at wetlandscape scale with determination coefficients greater than 0.57 in 7 out of 10 wetlandscapes. Our methodology and presented results may be used to map inundation seasonality and consequently account for its impact on wetland functions.

## 1. Introduction

Wetlands provide a multitude of ecosystem services (Mitsch and Gosselink 2000, Bullock and Acreman 2003, Zhu *et al* 2017), including groundwater recharge (McCarthy 2006, Freitas *et al* 2019, Wossenyeleh *et al* 2021), flood control (Hey and Philippi 1995, Watson *et al* 2016, Thorslund *et al* 2017), methane emission (Bloom *et al* 2017, Zhang *et al* 2017, Hondula *et al* 2021), storage (Lane and D’Amico 2010), provisioning of habitat for aquatic plants and animals (Knight *et al* 2001, Zedler and Kercher 2005, Benson *et al* 2018), and water quality buffering (Gilliam 1994, Sawatzky and Fahrig 2019) through the removal of carbon (Reuter *et al* 1992, Baptista *et al* 2003, Kayranli *et al* 2010), metals (Mungur *et al* 1995, Marchand *et al* 2010, Schück and Greger 2020, Sarkar *et al* 2021), sediments (Jordan *et al* 2003, Liu *et al* 2019, Wang *et al* 2019), and nitrate (Lin *et al* 2002, Golden *et al* 2017, Cheng *et al* 2020). Geographically Isolated Wetlands or GIWs (Tiner 2003a, Mushet *et al* 2015) are a specific subset of wetland systems that also provide the aforementioned services (Marton *et al* 2015, Calhoun *et al* 2017, Cheng and Basu 2017, Lane *et al* 2018), but have unfortunately been accorded limited protections (Creed *et al* 2017). This is partly because these wetlands are completely surrounded by uplands, and so they do not seem to be connected to other wetlands or waterbodies through a well-defined surface water connection, resulting in them being left out of jurisdictional reach. However, these wetlands can have hydrological connections with other waterbodies via subsurface flow or groundwater or may even have temporary surface water connections (Tiner 2003b).

The functions provided by GIWs are often related to their inundation characteristics (Melton *et al* 2013, Cohen *et al* 2016, Cheng and Basu 2017). The majority of the studies that quantify ecosystem services provisioned by wetlands (Bloom *et al* 2017, Zhang *et al* 2017, Hill *et al* 2018, Holmquist *et al* 2018) use a fixed area, which is often derived from National Wetlands Inventory (NWI) (U.S. Fish and Wildlife Service 2019) or other similar databases. This is despite the fact that wetlands exhibit intra-annual and inter-annual dynamics in their inundation characteristics, which in turn is bound to affect the services offered by them. While several recent studies have attempted to map regional wetland inundation and changes in it over the years (Huang *et al* 2014, Halabisky *et al* 2016, Jin *et al* 2017, Wu *et al* 2019), there has not been any comprehensive study to map out the seasonality of inundation for the primary wetlandscapes in the continental United States (CONUS).

This study addresses the aforementioned need by deriving the seasonality of GIWs across the CONUS for the first time. We also assess the change in seasonality over the analysis period. Consistency of the seasonality, i.e. the recurrence frequency of wet and dry months, is also evaluated. Finally, we estimate the degree to which the inundation seasonality can be explained based on monthly regional hydroclimatic forcings. To these ends, section 2 provides details of study sites and the method to delineate the time-series of the inundated area of GIWs. Section 3 presents the results regarding the seasonality of inundated areas of GIWs, its consistency, and meteorological controls on it. Section 4 summarizes the major results and conclusions, discusses the limitations, and suggests future research directions.

## 2. Data and methods

### 2.1. Data used to map wetland inundation

Remote sensing provides the opportunity to map spatio-temporal variations in wetland extent over large regions. Several efforts have been made in this regard, especially at local scales (Wu and Lane 2016, Hird *et al* 2017, Kaplan and Avdan 2018, Rezaee *et al* 2018, Wu *et al* 2019). Recently, the Global Surface Water (GSW; Pekel *et al* 2016) and Dynamic Surface Water Extent (DSWE; Jones 2015, 2019) maps have been used to study inundation dynamics (Allen and Pavelsky 2018, Luijendijk *et al* 2018, Mentaschi *et al* 2018). Here, we used the GSW inundation data from March 1985 to October 2015 to map the seasonality of inundated areas of GIWs. GSW was used here as it provides the opportunity to extend the methodology to other places beyond the CONUS. In addition, it is easy to use, and provides an estimate of inundation for each pixel within a month. The GSW v1.0 data was downloaded using the Google Earth Engine.

### 2.2. Selection of study sites

**Figure 1.**
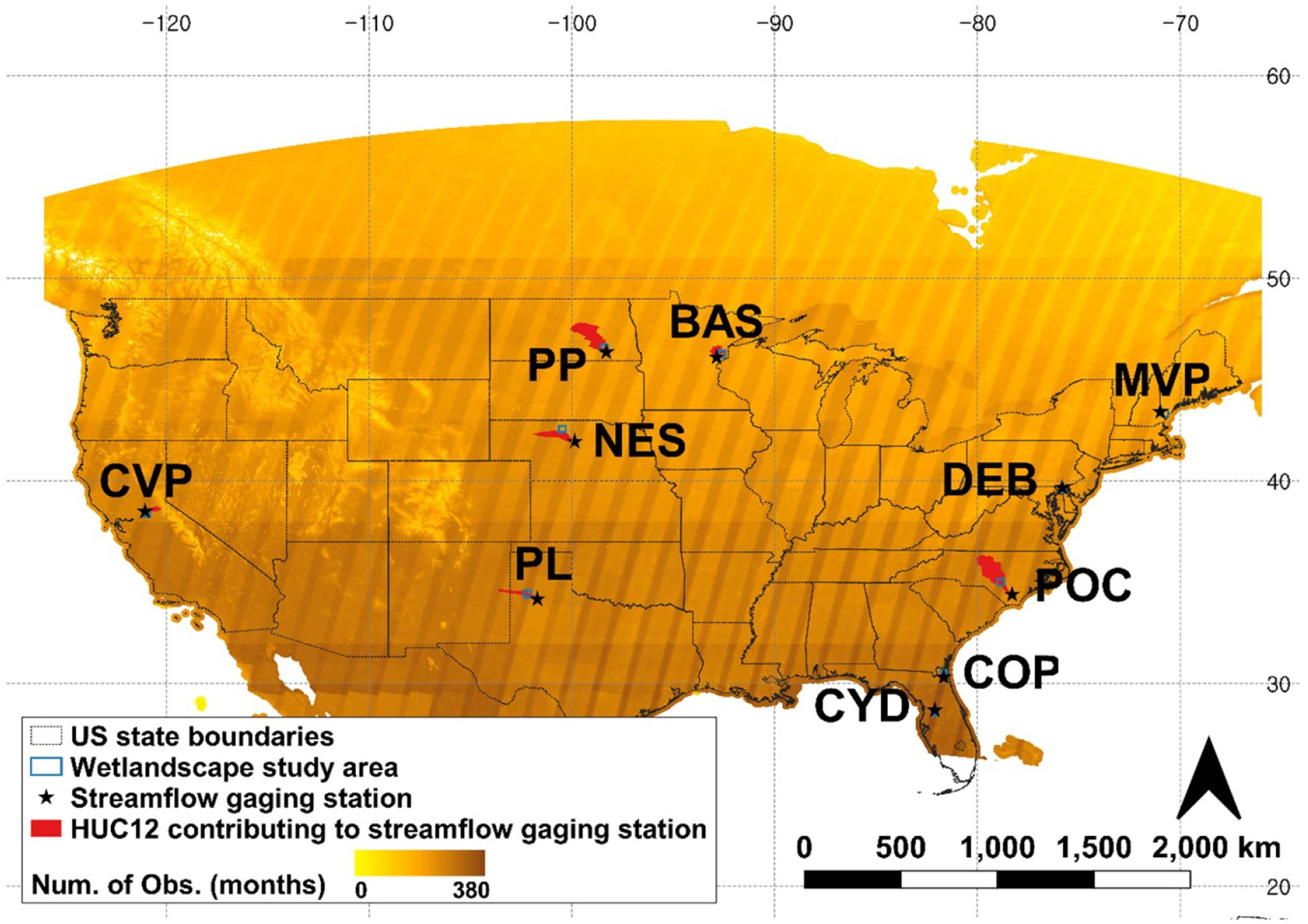
Rectangular regions (1000 km^2^ each) within different wetlandscapes within which inundation characteristics of geographically isolated wetlands are studied. These include the CVP, PP, BAS, MVP, PL, CYD, COP, POC, DEB, and NES. Also mapped are the number of monthly observations of Global Surface Water data (Pekel *et al* 2016) from March 1984 to October 2015. Locations of USGS gaging stations that are close to the selected wetlandscapes and their respective contribution areas are also shown. Zoom-in of the wetlandscapes and information regarding the USGS gaging stations are presented in figure S1 and table S1 of the supplementary information document, respectively.

We studied the inundation characteristics in ten large wetlandscapes of the CONUS (figure 1). These include the California vernal pools (CVP), Prairie potholes (PP), Basin wetlands (BAS), Maine vernal pools (MVP), Playa lakes (PL), Cypress domes (CYD), Coastal plain wetlands (COP), Pocosins (POC), Delmarva bays (DEB), and Nebraska Sandhills (NES). Inundation characteristics were assessed within a selected rectangular region of 1000 km^2^ area at each wetlandscape. The location of the rectangular region within each wetlandscape was determined based on the following heuristic: (a) wetlandscape locations were first identified based on the Cohen *et al* (2016) and Tiner (2003b), (b) within each wetlandscapes, locations with a high density of wetlands based on NWI data were subset, (c) from these subsets, locations with little or infrequent gaps in GSW data were identified, (d) rectangular regions with a long-term (>5 years, figure S1 and table S1 available online at stacks.iop.org/ERL/17/054005/mmedia) USGS discharge gaging station nearby for which the contribution area largely lies in the wetlandscape, were preferred. Post-processing of inundation data (figure S7) shows that the shape index or the ratio of the total square root of wetland area to total wetland perimeter in many of these landscapes have changed during the analysis period, thus indicating anthropogenic influences (Van Meter and Basu 2015, Krapu *et al* 2018).

### 2.3. Hydroclimatic data

To assess the role of hydroclimatic fluxes on the seasonality of GIWs’ inundation area, monthly forcings data such as rainfall, SM, evapotranspiration (ET), and potential evapotranspiration (PET) were obtained from the NLDAS-2 data set (Xia *et al* 2012). The majority of these fluxes have been validated in a range of settings (Luo *et al* 2003, Pan *et al* 2003, Nan *et al* 2010, Nearing *et al* 2016) and has been used extensively in hydrologic studies (Mitchell *et al* 2004, Yu *et al* 2013, Maxwell *et al* 2015, Wang *et al* 2018, Zhang *et al* 2019). Data were extracted for all NLDAS-2 grids that intersected with 1000 km^2^ study region within each wetlandscape. Records corresponding to each wetlandscape were spatially averaged.

### 2.4. Identifying GIWs from NWI

**Figure 2.**
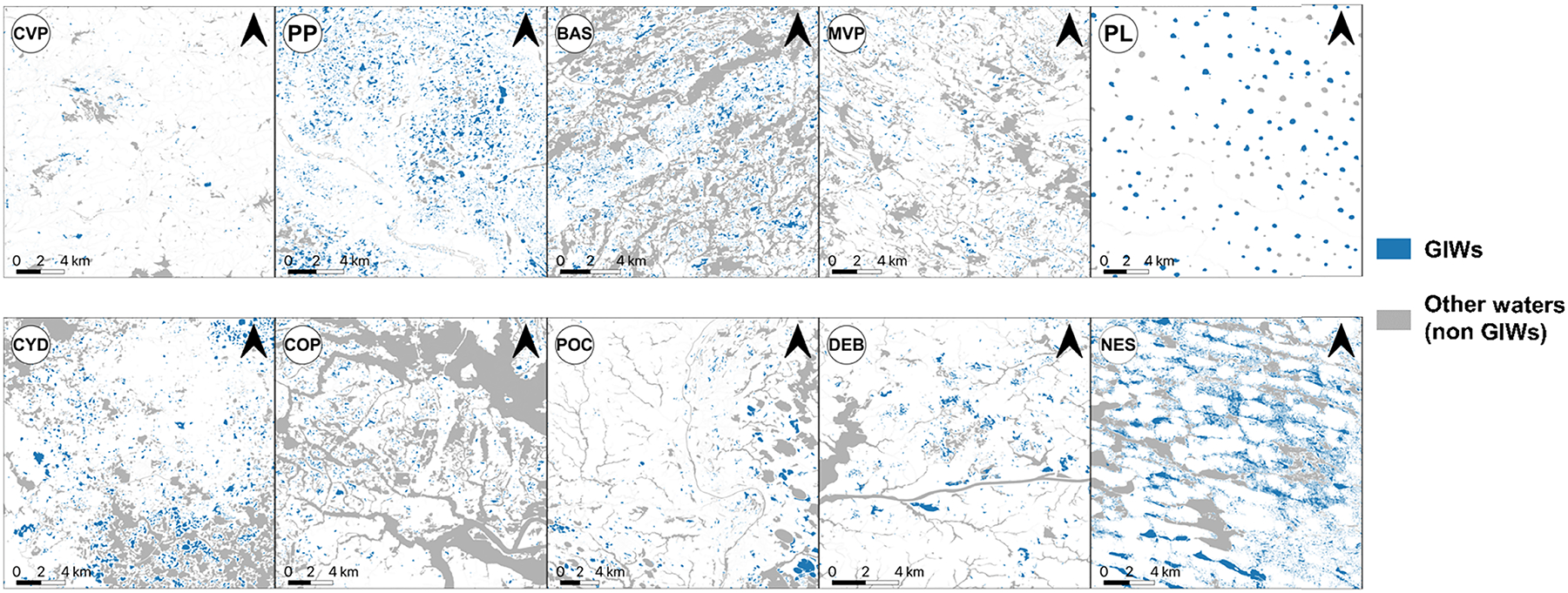
Geographically isolated wetlands (GIWs) and other National Wetland Inventory (NWI) wetlands in the study areas. Description of the wetlandscape acronyms are provided in figure 1 caption.

GIWs were derived within the selected rectangular regions. To this end, we used a method similar to the one presented in Lane and D’Amico (2016). The method involves using the NWI and National Hydrographic Dataset (NHD). To select GIWs from NWI, first riverine, marine, and estuarine wetland systems were excluded from the NWI, i.e. only the palustrine and lacustrine wetlands were considered for further analysis. Next, NHD data that includes hydrographic features such as rivers, lakes, reservoirs, wetlands, and oceans was used. Following Lane and D’Amico (2016), we obtained 10 m buffer polygons for rivers, NHD lakes larger than 8 ha, other water bodies (e.g. reservoirs, playa, etc) larger than 1.5 ha, and all other flowlines and area features (including bays/inlets, locks, levees, etc.). Palustrine and lacustrine wetland polygons, selected above from NWI, that intersect with the 10 m buffered NHD polygons were then removed, as they were considered to have surficial connections with other waterbodies. The remaining wetlands were termed as GIWs. The resulting extracted wetlands (figure 2) were the considered GIWs for ensuing analyses.

### 2.5. Delineating the dynamics of GIWs

**Figure 3.**
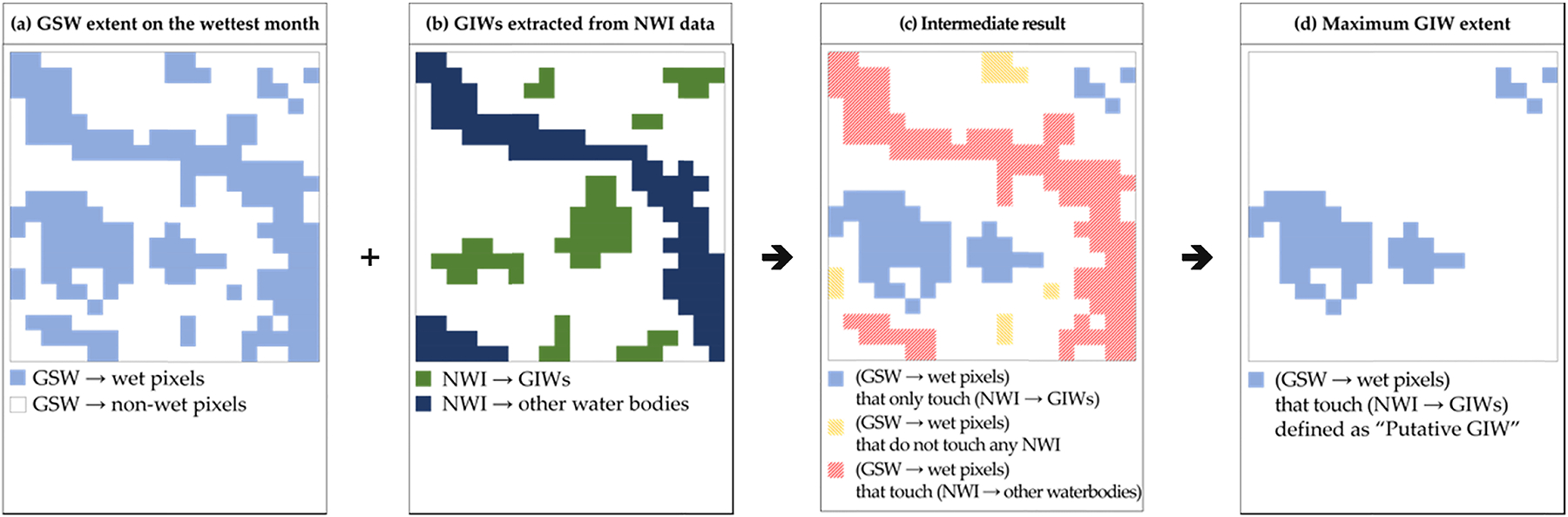
Procedural steps for delineating the maximum GIW extent from the wettest GSW month.

To evaluate GIW dynamics, first, the maximum extent of GIWs was derived from the GSW maps. To this end, a multi-step process was followed (figure 3). By counting all the water pixels in GSW data within a wetlandscape, the inundation area for every month from March 1984 to October 2015 was evaluated. From this, the wettest month during the analysis period, characterized by the maximum inundation area within a wetlandscape, was identified. For the wettest month in the GSW map, we then distinguished wet pixels into GIWs and non-GIWs. Non-GIWs were the contiguous wet pixels that touched or intersected with water bodies that are not classified as GIWs in the NWI data (identified in section 2.4) or, in other words, water bodies that are termed as NWI → other waterbodies in figure 2. If contiguous wet pixels did not touch any GIWs in the NWI data (or NWI → GIWs in figure 2), these pixels were classified as non-GIWs as they are likely to be either non-GIW wetlands or ephemeral pondings or misclassifications in the satellite data. It is also possible that commission errors were also due to poorer data resolution and inconsistent mapping protocol of older NWI data (Tiner 1997, Wu and Lane 2017), as we assumed the NWI to be the most accurate baseline dataset for these analyses. The rest of the wet pixels in the GSW map were identified as GIWs. Each contiguous wet pixel region was considered a GIW henceforth. The derived map represented the maximum extent of selected GIWs during the analyses period. It is to be noted that there is a possibility that the identified extent may not be the absolute maximum extent of the selected GIWs in reality, as the analyses could only be performed for the dates for which GSW data exists.

The maximum extent of GIWs, extracted above was then used to obtain the temporal dynamics. To this end, for each GIW, the inundation area was evaluated for all months, with no-data values being less than 5% of the maximum extent area. When no-data pixels exceeded 5% of the maximum extent area of a given GIW for a given timestep, the record was neglected. Next, the inundation area fraction (IAF) of a GIW was calculated as the ratio of the wet pixel area in a given timestep divided by the all-time maximum wet pixel area of the GIW. The IAF varies from 0 to 1, thus providing a standardized inundation fraction for GIWs. IAF equal to zero indicates a completely dry GIW or a GIW with no water detected within it. A value of one indicates its maximum extent. For many GIWs, the minimum is greater than zero, indicating that those GIWs do not fully dry up. During wet periods, it is expected that individual GIWs will have larger IAFs. Next, the average IAF for a given month overall GIWs within each wetlandscape, hereafter called allGIW_IAF, was obtained. Finally, mean monthly allGIW_IAF (allGIW_IAF_m_ hereafter) and monthly standard deviation of allGIW_IAF (allGIW_IAF_std_ hereafter) were evaluated. In other words, a single allGIW_IAF_m_ for January was obtained based on allGIW_IAF estimates from all January months in the data period. The intra-annual variation in allGIW_IAF_m_ indicates the seasonality of GIW dynamics within a wetlandscape, while allGIW_IAF_std_ provides a measure of inter-annual variations in averaged monthly inundation fractions.

### 2.6. Filling gaps in the estimate of mean and std. deviation of GIW inundation area

**Figure 4.**
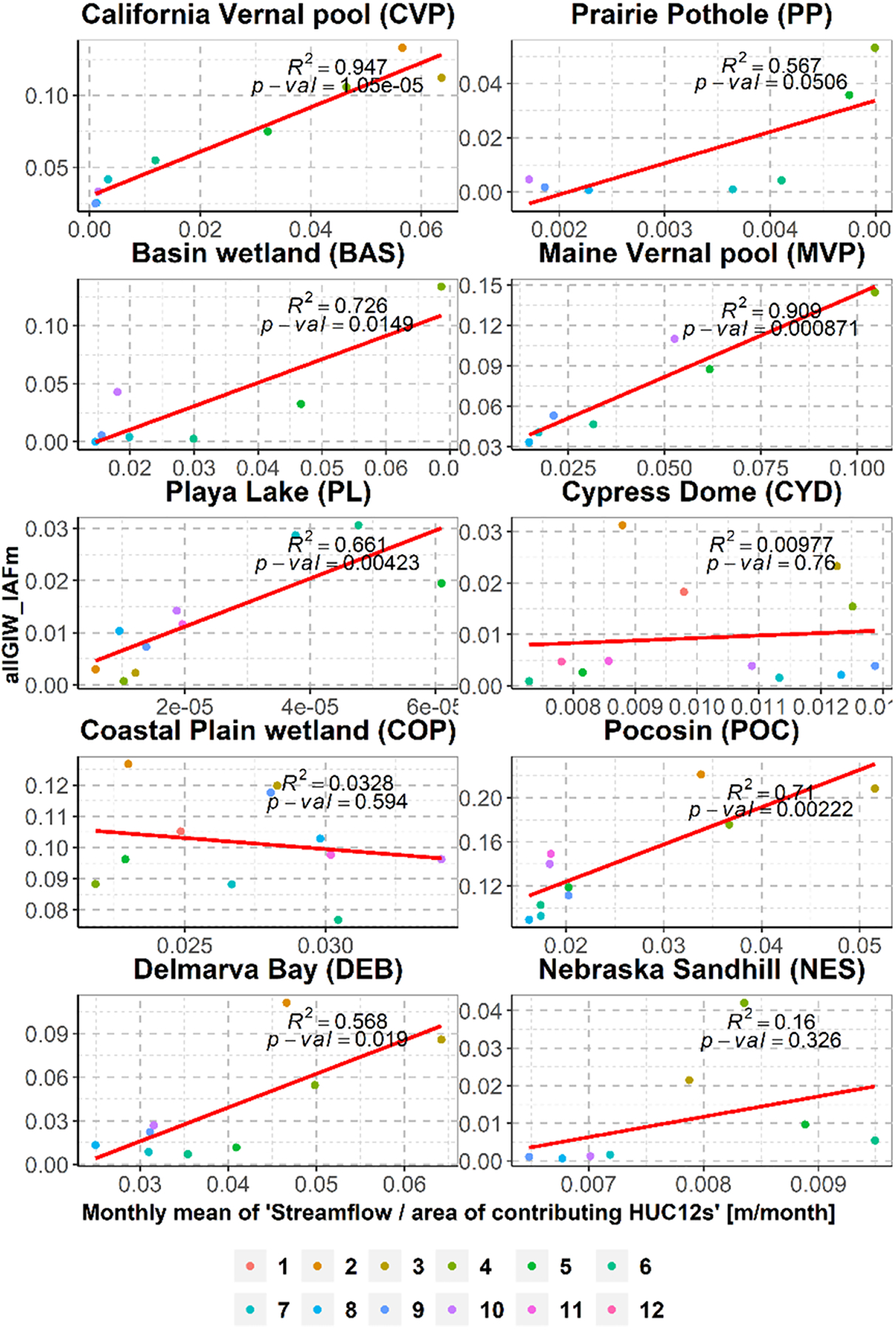
Scatter plot between the monthly mean of averaged inundated area fraction over all GIWs (allGIW_IAF_m_) and monthly mean of streamflow rate per unit area at the streamflow gaging stations (in m/month). The straight line is the linear regression fit. Months 1–12 indicate January–December.

For certain months and locations, GSW data are non-existent (see figures 1 and S2). Following cues from recent studies (e.g. Walker *et al* 2020) that have demonstrated a correspondence between wetland area and regional water availability descriptors such as streamflow, regressions between inundation characteristics, and monthly streamflows were developed. Here, the allGIW_IAF_m_ (or allGIW_IAF_std_) for the months with data were considered the response variables. The explanatory variable was the mean monthly streamflow per unit contribution area at a nearby streamflow gaging station. It is to be noted that other meteorological variables such as precipitation (SM + rain) and (precipitation—ET ) were also used to generate these relations, but streamflow turned out to be the most explanatory variable overall (see table S2). More information regarding the contribution area of streamflow gaging stations is provided in figures 1, S1 and table S1. Using the regression equations derived in figures 4 and S3, estimates of allGIW_IAF_m_ (allGIW_IAF_std_) for the months with missing data were obtained. The filling was performed in wetlandscapes CVP, MVP, PL, POC, and DEB. For the wetlandscapes where the regression performance was not satisfactory, i.e. with *p*-value > 0.05 (e.g. wetlandscapes COP and NES), the filling was not performed. As the goal of this study is restricted to assessing the seasonality of wetlands, instead of the exact magnitude of allGIW_IAF_m_ (or allGIW_IAF_std_), we qualitatively evaluated our overall results with respect to observation data from isolated observations in the considered wetlandscapes. Notably, an alternative regression configuration was also considered. Herein, the response variable was the average inundation fraction of all GIWs for each time step or allGIW_IAF, and the explanatory variable was the corresponding streamflow for these time steps. Results from the analyses indicate that the selected regression configuration (shown in figure 4) is overall more effective than one shown in figure S4 and hence was used for the ensuing analyses.

### 2.7. Assessing the role of hydroclimatic forcings on inundation seasonality

Inundation dynamics of GIWs are expected to depend on a range of water exchange fluxes (figure S6). GIWs are often recharged by falling rain and snow on them or by the fluxes indirectly supported by rain and SM, such as lateral groundwater flow between wetland and the neighboring aquifer (Neff *et al* 2020, Park *et al* 2020) and runoff from the local contributing area (Shook *et al* 2013, Wang *et al* 2021). Notably, runoff from the contributing area is also a function of rain, SM, and ET. Water is removed from wetlands by open water evaporation, ET via vegetation, and bed leakage (Hayashi *et al* 2003, Shook *et al* 2013, Liu and Kumar 2016). While fine spatio-temporal resolution estimation of these fluxes for each wetland at the scale of our analysis is prohibitive due to lack of refined data and prevalence of a range of processes in play, the overall seasonality is expected to be a function of rain (R), PET, ET, and snowmelt (SM) (Sánchez-Carrillo *et al* 2004, Park *et al* 2014, Penatti *et al* 2015, Liu and Kumar 2016, Zhu *et al* 2017, Bertassello *et al* 2020, Lee *et al* 2020). Here our goal is to assess the extent to which regional data of R, PET, ET, and SM, with inherent uncertainties, can explain the seasonal dynamics of GIW inundation at wetlandscape scale.

To assess the extent to which hydroclimatic forcings influence the seasonal wetland dynamics, multiple linear regression with variable selection was performed at each wetlandscape. The change in allGIW_IAF between consecutive months or allGIW_IAF(t)-allGIW_IAF(t-1) (hereafter ΔallGIW_IAF) was the dependent variable. Here t is the index for a given month. Use of ΔallGIW_IAF, instead of allGIW_IAF, as the dependent variables were driven by the fact that similar allGIW_IAF could be observed within a year during seasonal recharge and recession periods when the meteorological forcings could be very different. The candidate independent variables were R, ET, PET, and SM. For the analysis period of *N* years, regressions were generated using different combinations of data of *N-1* years based on the leave-one-out method. All 15 possible subsets of independent variables were considered one-by-one. Among the subsets of independent variables, the subset producing the highest *R*^2^ was selected. This subset was then used to predict ΔallGIW_IAF for the year not considered in the regression. The process was repeated for other *N-1* years. This was followed by evaluation of allGIW_IAF(t) for all months during a year, for every year, using the equation allGIW_IAF(t) = allGIW_IAF(t-1) + ΔallGIW_IAF(t). Predicted allGIW_IAF for all years were then averaged to obtain the seasonality in allGIW_IAF_m_.

## 3. Results and discussion

### 3.1. Seasonality in inundation area of GIWs

**Figure 5.**
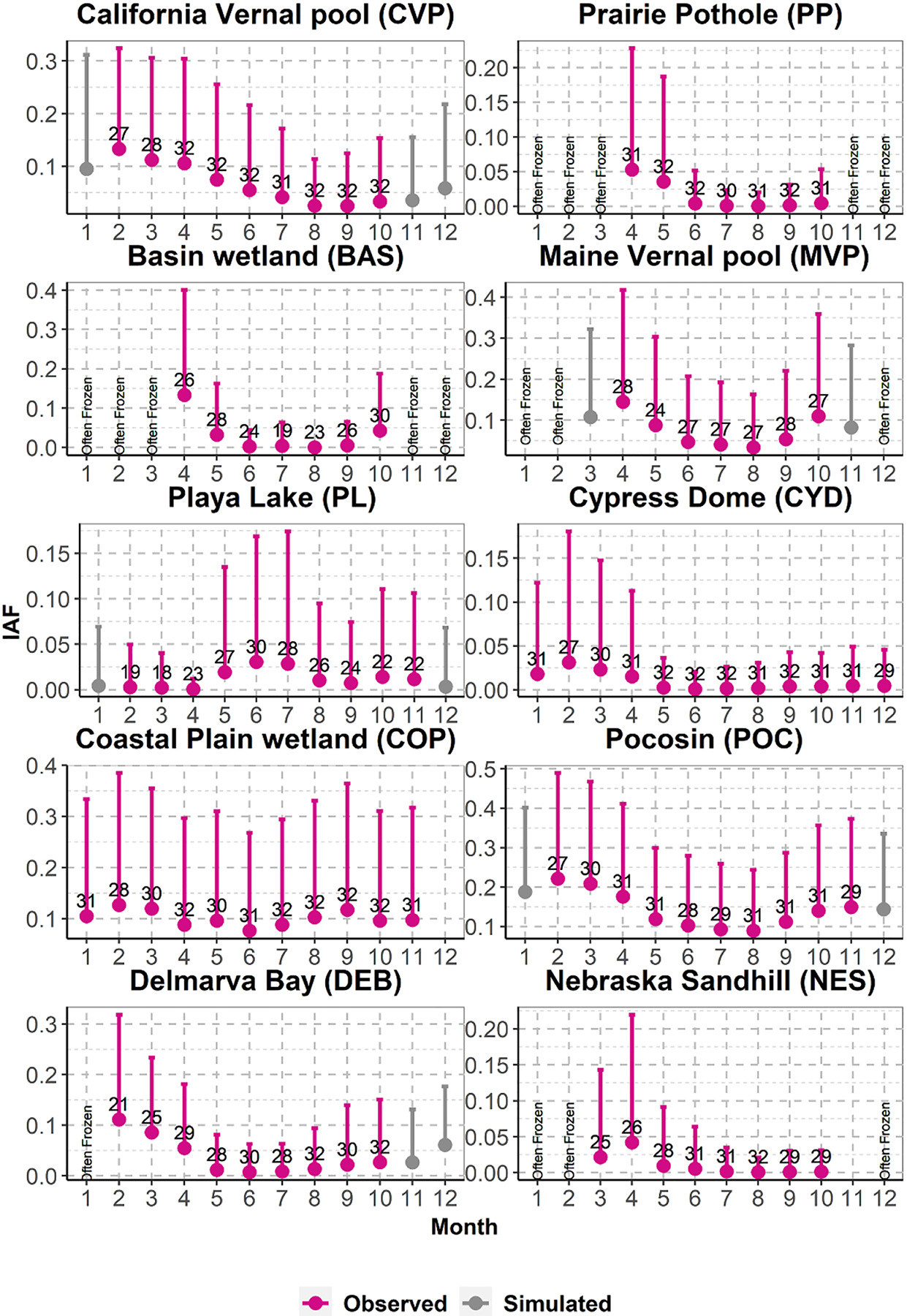
Box plot of monthly averages and standard deviations of IAF. Red dots and whiskers are obtained using observation data. Estimates for months for which data does not exist are shown in grey. These estimates were obtained using the linear regression models shown in figures 4 and S3. Dots indicate monthly averaged inundated area fraction over all GIWs (allGIW_IAF_m_), and the top extent of whiskers indicate the allGIW_IAF_m_ + allGIW_IAF_std_. Numbers written above the red dots indicate the number of years for which IAF data is available in the concerned month.

Intra-annual variations of IAFs in wetlandscapes are shown in figure 5. Overall, wetlands that are affected by SM (hereafter SM-affected), such as the PP, BAS of Minnesota, MVP, and NES, experienced peak allGIW_IAF_m_ in April. In these wetlandscapes, wetlands may often be frozen in certain winter months. Here the inundation area evaluation is performed only for the months with average air temperature greater than zero in at least 50% of the observation years. It is to be emphasized that the shown temporal variations do not indicate that all GIWs in these wetlandscapes were at their largest inundation extent in April every year, but rather the mean of monthly extents over all GIWs are maximum in April. After April, allGIW_IAF_m_ progressively decreased till August. Following August/September, the decreasing trend usually reversed. The extent of intra-annual variations in allGIW_IAF_m_, however, showed differences across wetlandscapes. For example, compared to the maximum allGIW_IAF_m_, the minimum allGIW_IAF_m_ for PP, BAS, MVP, and NES were 1.4%, 0.0%, 23.1%, and 1.6% in magnitude. The coefficient of variation or the ratio of standard deviation to the mean, indicating the magnitude of intra-annual variations of allGIW_IAF_m_, for these wetlands were 1.46, 1.51, 0.56, and 1.40, respectively. Notably, intra-annual variation of allGIW_IAF_m_ in MVP was found to be relatively smaller than in other wetlandscapes. Recovery of the wetland area in fall was also the highest in the MVP, with October allGIW_IAF_m_ being around 76% of the maximum allGIW_IAF_m_.

Seasonal variations obtained above, for the most part, align with the groundwater table or water depth variations observed at isolated gaging stations within the concerned wetlandscapes. For example, in the PP region, Johnson *et al* (2004) observed peak water table elevation during April/May and a decrease in the following months. In some years, such as the deluge years of 1994 and 1995 in their observation period, they also reported a second peak in mid-summer or early fall. In most years, the decreasing trend reversed in September/October. It is to be noted that while our results indicate an increase in variability in mid-summer, the data does not show a significant increase in allGIW_IAF_m_ during this period. Observation sites in BAS and NES also corroborate the overall reported intra-annual variations of allGIW_IAF_m_ at these wetlandscapes, with peak timing in April followed by a reduction in summer and subsequent recovery in fall. For example, BEC (2010) and MBWSR (2013) reported the highest water table during April/May, a decreasing trend in summer, and recovery in fall in wetlands within Saint Louis and Dodge counties of Minnesota.

Similarly, Gosselin *et al* (1999) and Gosselin *et al* (2000) observed higher water elevations during March to May within the sandhills of Nebraska. While studies detailing water table dynamics for vernal pools in Maine are scant, its description as ‘small, ephemeral wetlands that typically fill in spring with SM and precipitation, or fall with rising water tables, and are dry by summer’s end’ aligns with the reported temporal variation of inundation area in figure 5 (Calhoun *et al* 2014, p 11 002). Kifner *et al* (2018) also described MVP as ‘typically at their highest water level in the spring, dry down by mid-summer, and refill in the autumn, while some dry on cycles longer than a year.’

Among the wetlands that are minimally affected by SM or, in other words, those mostly driven by rain (hereafter rain-fed), the intra-annual variations of inundation were generally similar to each other, but some did show stark differences (figure 5). For example, the allGIW_IAF_m_ peaked in February in CVP, Florida CYD, COP, POC of North Carolina, and Delmarva bays in Maryland (DEB). In contrast, the peak of allGIW_IAF_m_ in PL of Texas was in June. Except for PL, all rain-fed wetlandscapes experienced a decrease in allGIW_IAF_m_ in the months following February, with minimum allGIW_IAF_m_ usually occurring during May to August. COP and POC showed a second smaller peak during fall, with allGIW_IAF_m_ magnitude being around 93% and 68% of its maximum value, respectively. In PL, the minimum allGIW_IAF_m_ usually occurred between February to April. In terms of the extent of variations, the ratio of minimum to maximum allGIW_IAF_m_ for CVP, CYD, COP, POC, DEB, and PL were 18.7%, 3.1%, 60.6%, 40.4%, 6.5%, and 2.5%, respectively. The coefficient of variation of allGIW_IAF_m_ for these wetlands were 0.61, 1.07, 0.15, 0.33, 0.99, and 0.82, respectively. The result indicates that the intra-annual variation of the allGIW_IAF_m_ was relatively moderate in POC and DEB.

Overall, the intra-annual variations in rain-fed wetlands reported in figure 5 align with observations at isolated sites in these wetlandscapes, albeit with a few exceptions. For example, Rains *et al* (2006) observed significant variations in stages in vernal pools following precipitation events, with an overall decreasing trend from February to June. Similarly, Brown (1981) and Kasischke *et al* (2003) reported a water level peak in January in CYD of Florida. Notably, these studies also noted additional isolated peaks between August and October in response to intense precipitation that oftentimes originated from hurricanes or tropical storms. Along the lines of reported results for COP shown in figure 5, Williams *et al* (2002) and Amatya *et al* (2020) observed water table depths in wetlands near the southeastern Atlantic coast peak in spring and autumn and be relatively dry in summer. The distinct seasonality of inundation in PL (see figure 5) is in line with the water depth observations reported by Ganesan (2010) and Weinberg *et al* (2015), where water table peaks were reported in July/August with smaller values in fall and winter.

**Figure 6.**
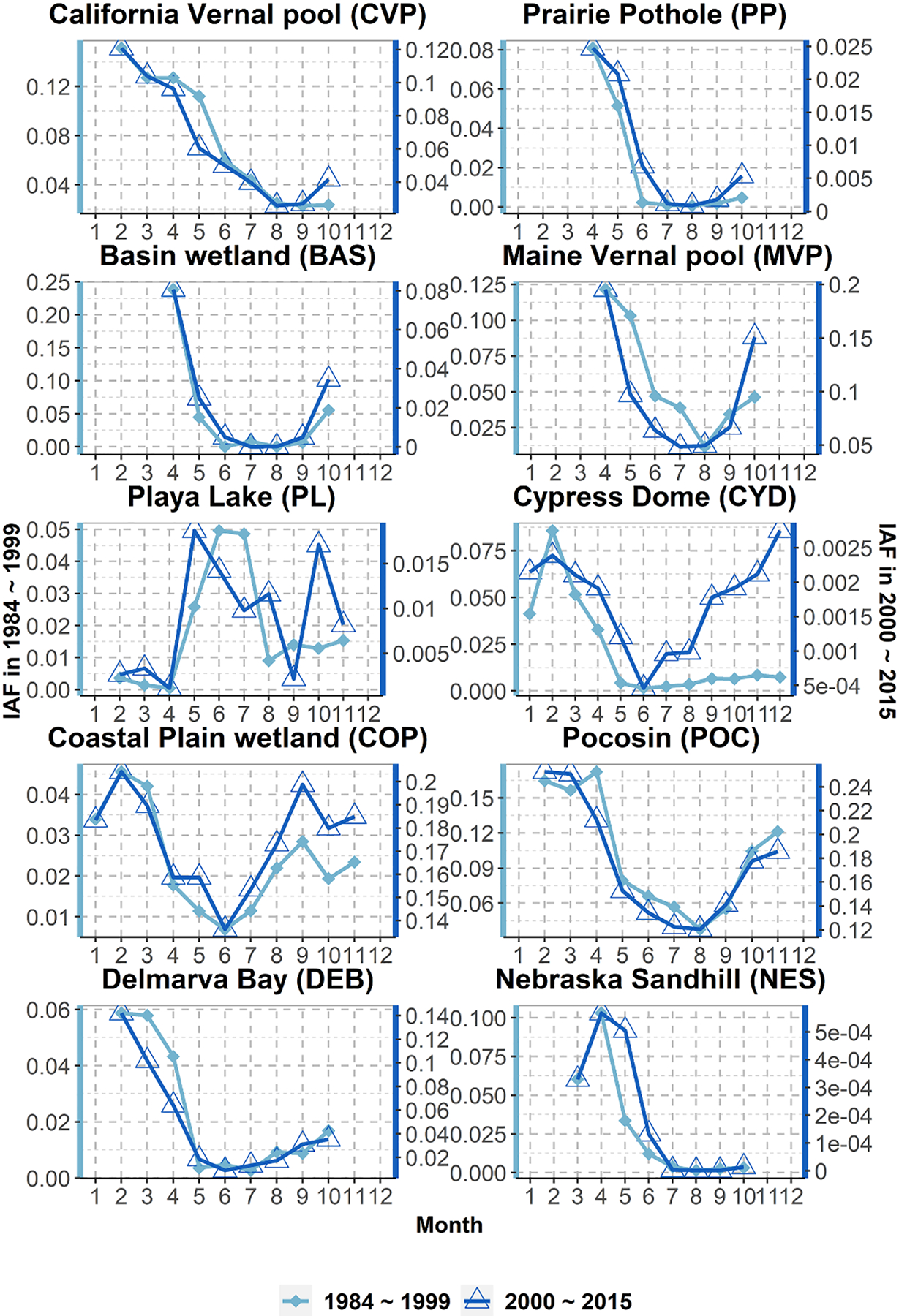
Changes in long-term allGIW_IAF_m_ between the two halves of the analysis period (1984–2015). Note that the *y*-axes ranges for the two periods are different.

Overall, during the analyses period, the long-term seasonality or the intra-annual variation of allGIW_IAF_m_ showed changes in terms of the inundation extent (figure 6). Following the strategy used in Borja *et al* (2020), we divided the analyses period into two halves to track the long-term change. Seasonality was then obtained for both these periods. Eastern CONUS wetlandscapes, including MVP, COP, POC, and DEB, showed an overall increase in wetland extent in the later period. In contrast, western wetlandscapes such as CVP and NES showed an overall decrease in inundation extent. These results are consistent with previously reported open surface water trends (Zou *et al* 2018, Borja *et al* 2020), although it is to be noted that our analyses capture the changes in the inundation extent of the same GIWs over time while the previous studies evaluated the total open surface area over a landscape. This could possibly be the reason why our results show a decrease in inundation, especially during the spring melt, in BAS and PP wetlandscapes, which is in contrast to the results reported in Borja *et al* (2020). Notably, even though the IAFs for these wetlandscapes have changed over time, the dry and wet periods during the year have remained almost the same. For PL, the month with the largest and smallest allGIW_IAF_m_ shifted by a month.

### 3.2. Consistency of seasonality in inundated area of GIWs

The previous section discussed the seasonality or the intra-annual variation of allGIW_IAF_m_ for each wetlandscape. However, as IAF shows significant variations from one year to next (see the monthly mean standard deviation shown in figure 5), it is expected that the maximum (minimum) allGIW_IAF in a given year may not occur in the month with maximum allGIW_IAF_m_. To assess inter-annual variations in inundation seasonality, its consistency, quantified by the fraction of years the maximum (minimum) allGIW_IAF occurs in the top three (bottom three) months of allGIW_IAF_m_, was evaluated. For calculating the consistency, only the years with at least one record among the top three or the bottom three months were used.

**Figure 7.**
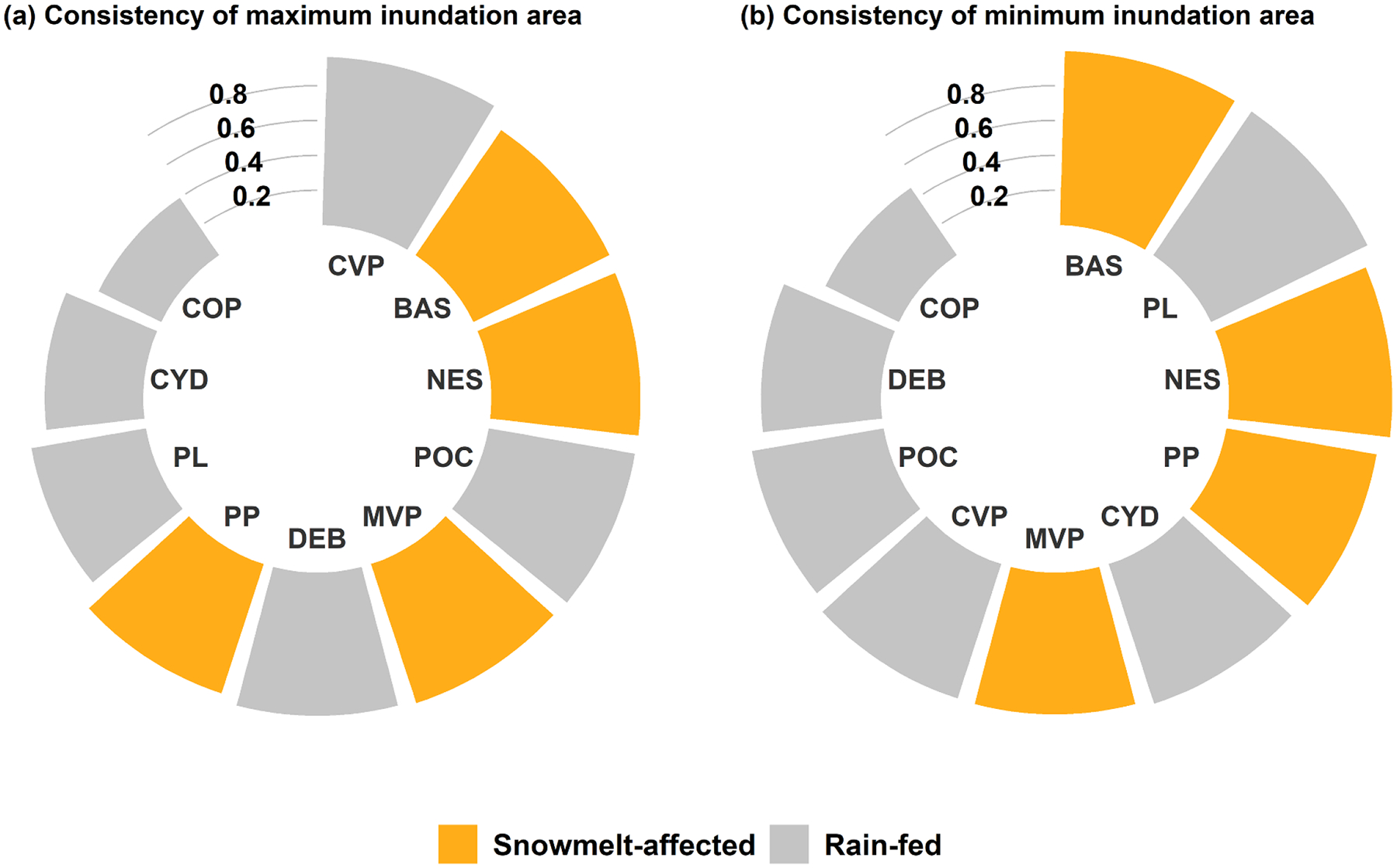
Consistency of seasonality quantified as the fraction of years the maximum or minimum IAF occurs in the top three or bottom three months, respectively, in terms of the magnitude of allGIW_IAF_m_. Description of the wetlandscape acronyms are provided in figure 1 caption.

In most wetlandscapes, the consistency of month with the maximum inundation area was more than 50% (figure 7), i.e. in more than half of the years, the maximum inundation area lies within the top three months in terms of the magnitude of allGIW_IAF_m_. For SM-affected wetlandscapes, i.e. PP, BAS, MVP, and NES, corresponding consistencies were all greater than 75%. High consistency in SM-affected wet-lands is unsurprising to some extent, as the SM is dominantly driven by annually recurring higher temperatures and radiation in late spring and early summer. Arguably, an even higher consistency should perhaps be expected for SM-affected wetlandscapes. However, this is not so due to a few large rainfall events in late summer and fall in some of these wetlandscapes, as noted in Neill (1993) and Johnson *et al* (2004), which may cause widespread inundation. The consistency of month with the maximum inundation area was relatively smaller for rain-fed wetlandscapes CYD and COP, with consistency magnitude being smaller than 50%. Notably, both CYD and COP experience occasional flooding due to hurricanes and/or tropical storms during June to November, thus leading to seasonal peaks in months that do not coincide with the three months with mean maximum allGIW_IAF_m_. The lower consistency of the month with minimum allGIW_IAF_m_ for COP can again be partially attributed to these storms. Overall, SM-affected wetlandscapes experience high consistency for the month with minimum allGIW_IAF_m_, in part due to distinct seasonality of net soil water input that is dominated by SM in late spring/early summer and relatively higher ET rates in summer.

### 3.3. Hydroclimatic drivers of seasonality

Qualitatively, the monthly variation of both long-term average R + SM and R + SM-ET (see figure S5) shows some correspondence with the intra-annual variation of allGIW_IAF_m_ (figure 5 and table S2). Notably, at least one variable between long-term average R + SM and R + SM-ET with lag of 0 and 1 month shows a coefficient of determination or r^2^ value of more than 0.5 in seven out of ten sites. The other three sites where *r*^2^ < 0.5 are CYD, COP, and POC. All of these three sites are affected by rapid inundation due to hurricanes and tropical storms. Overall, R + SM showed better *r*^2^ to allGIW_IAF_m_ at more sites than R + SM-ET. The aforementioned results indicate that long-term average R + SM and/or R + SM-ET show similar intra-annual variations as allGIW_IAF_m_ for most sites.

Next, we quantitatively assess the extent to which inundation seasonality can be predicted by the seasonality of controlling fluxes. Here the assessment was performed for the two halves of the analysis period, viz. 1984–1999 and 2000–2015, using the methodology outlined in section 2.7. The reason to do the separate assessment for the two periods is that smaller period constraints the extent of anthropogenic change, if any, thus allowing a better understanding of the influence of controlling fluxes on inundation seasonality. Results (figure S8 and table 1) indicate that regressions based on R(t), SM(t), PET(t), and ET(t) can capture more than 52% (57%) of the intra-annual variations in allGIW_IAF_m_ in 8 out of 10 wetlandscapes during 1984–1999 (2000–2014).

## 4. Summary and conclusions

The study evaluated, for the first time, the seasonality of wetland inundation in the major wetlandscapes of the CONUS. This was made possible through the development of an approach to obtain the long-term intra-annual variation of inundation or allGIW_IAF_m_ in different wetlandscapes, even as the source data of surface inundation had numerous gaps in both space and time. Notably, the approach presented here may be used to map inundation seasonality in alternative wetlandscapes across the globe. Next, the study also evaluated the consistency of intra-annual variations and changes in them during the analyses period. Finally, an assessment of the extent to which the seasonality can be explained based on meteorological fluxes was assessed.

Overall, SM-affected vs. rain-fed wetlandscapes showed a stark difference in the seasonality of inundation. The top three wetlandscape in terms of the coefficient of variation in allGIW_IAF_m_ were SM affected, indicating large changes in average inundation extent w.r.t. its mean during the year. In contrast, the POC and DEB, both rain-fed wetlands, showed the smallest coefficient of variation in allGIW_IAF_m_. SM-affected wetlandscapes such as PP, BAS, MVP, and NES are generally found to be maximally inundated during April following spring melt, and then they reduce in the area till August/September when the trend reverses. The derived seasonality is generally found to qualitatively align with observation data from isolated wetlands in the considered wetlandscapes. It is to be noted that some differences in the derived intra-annual variation of allGIW_IAF_m_ and previously reported variations in water table depth that were observed at isolated wetlands are expected. This is because allGIW_IAF_m_ provides an aggregated inundation characteristic of multiple wetlands located within 1000 km^2^ area over 31 years, while water table depth observations are generally available for only a few wetlands and that too for a limited period. Another source of discrepancy could be the coarse spatio-temporal resolution of the GSW data, which is only available once a month and at a spatial resolution of 30 m.

Despite the anthropogenic and climatic changes that the considered wetlandscapes may have experienced during the analysis period (1984–2015), they exhibited minimal changes in the timing of dry and wet periods. In terms of the inundation extent, however, contrasting changes in inundation magnitudes were observed. The consistency of the seasonality, a metric to capture the frequency with which the maximum (minimum) inundation occurs in the top three (bottom three) months as defined by long-term averaged inundation area, was generally high in SM-fed wetlands. Even though the rain-fed wetlandscapes are distributed from coast to coast, the majority of them showed remarkable similarity in their seasonality. For example, CVP, CYD, COP, POC, and DEB had the maximum monthly average inundation in February and a minimum during June/August. COP and POC, however, did show a second peak during fall. An exception in seasonality for rain-fed wetlands is the PL, which exhibits maximum monthly inundation in June and minimum during February to April. The inter-annual consistency of the derived seasonality was relatively poor in PL, CYD, and COP, in part due to relatively moderate variations in monthly averaged inundation area across months and the existence of short-burst precipitation events. Overall, widely available data of regional hydroclimatic fluxes were able to explain more than 57% of the seasonality in 7 out of 10 wetlandscapes. The result indicates that despite the numerous hydrologic processes and wetland attributes that determine inundation dynamics, simple multiple linear regressions using widely available meteorological fluxes can capture inundation seasonality at the wetlandscape scale. Similar regressions may be derived for alternative wetlandscapes across the globe, and if they are found to be sufficiently representative across periods, they may be used for assessment of changes in inundation seasonality with changes in climate (Zhu *et al* 2017). These regressions could also be used to fill allGIW_IAF_m_ estimates for months with insufficient data, as explored in section 2.6.

Although significant care was taken in data analyses, and only GIWs with less than 5% missing data were chosen for analyses, some uncertainty in our conclusions regarding the seasonality could have been introduced by (a) missing GSW data in certain months (see figures 5 and S2), (b) presence of clouds and canopy, (c) use of coarse temporal (once a month) and spatial resolution (30 × 30 m) of the data, (d) inherent uncertainty in the GSW product, (e) presence of barriers that could partition GIWs, (f) uncertainty in regressions that are used to fill mean monthly inundation fraction (allGIW_IAF_m_) in months with missing/insufficient data, and (g) undefined date of data capture used to identify inundation pixels. Recent satellite missions such as Sentinel-2 (Wang and Atkinson 2018), WorldView-3 (Pacifici *et al* 2015), SuperView-1 (Kuang *et al* 2018), and those by commercial platforms such as Planet Lab (www.planet.com/) with high-resolution images and advanced quality control processes may help reduce few of these uncertainties to an extent.

Despite the aforementioned challenges, this study, for the first time, maps the seasonality of GIWs, and the changes in it, within the major wetlandscapes of the CONUS using long-term (>30 years) data. In addition, it delineates the inter-annual consistency of the reported seasonality and highlights the role of meteorological controls on them. Given the role of wetlands’ inundation on numerous ecosystem services, as outlined in section 1, results of this study and the methods developed herein to map wetland seasonality may be used to assess within-year inundation dynamics’ impacts on these services.

## Supplementary Material

Sup 1

## Figures and Tables

**Table 1.** Performance of the best multiple linear regression after variable selection using leave-one-out approach (as outlined in section 2.7) between allGIW_IAF_m_ and monthly averaged ET, PET, R (rainfall), and SM. Description of the wetlandscape acronyms are provided in figure 1 caption.

Wetlandscapes	*R* ^2^	
	1984–1999	2000–2015
CVP	0.84	0.98
PP	0.90	0.90
BAS	0.52	0.58
MVP	0.93	0.77
PL	0.12	0.46
CYD	0.93	0.86
COP	0.88	0.63
POC	0.37	0.51
DEB	0.83	0.57
NES	0.87	0.85

## Data Availability

Codes will be made available upon reasonable request from the authors.
