# Supplementary material for: Seasonality of inundation in geographically isolated wetlands across the United States
Source: Environ Res Lett. Author manuscript; Available in PMC 2022 Jun 2. (PMC9161429; doi:10.1088/1748-9326/ac6149)

Table S1. The ID of USGS gaging stations for the selected wetlandscapes.

Wetlandscapes	ID of USGS stream gaging station	Location	Available monthly streamflow data
**CVP**	USGS 11335000	38°30'01", 121°02'39"	Mar 1984 ~ Oct 2015
**PP**	USGS 06470500	46°21'20", 98°18'15"	Mar 1984 ~ Oct 2015
**BAS**	USGS 05336700	46°06'20", 92°51'50"	Mar 1984 ~ Oct 2015
**MVP**	USGS 01072100	43°24'48", 70°59'15"	Mar 1984 ~ Jun 2005
**PL**	USGS 08080700	34°10'44", 101°42'08"	Oct 2002 ~ Oct 2015
**CYD**	USGS 02312640	28°41'49", 82°06'15"	Mar 1984 ~ Oct 2015
**COP**	USGS 02246500	30°19'20", 81°39'56"	Jul 1987 ~ Sep 1988<br>Jan 1989 ~ Nov 1989<br>…<br>Feb 2015 ~ May 2015<br>Jul 2015, Sep 2015
**POC**	USGS 02105769	34°24'16", 78°17'37"	Mar 1984 ~ Oct 2015
**DEB**	USGS 01495000	39°40'03.3", 75°49'32.1"	Mar 1984 ~ Oct 2015
**NES**	USGS 06785500	41°56'30", 99°51'37"	May 2010 ~ Oct 2015

Table S2. Coefficient of determination (r^2) between allGIW_IAF$_m$ and hydroclimatic variables at different lags.

	r^2 2					
**Wetlandscapes**	**allGIW_IAFm vs. Streamflow [lag 0 months]**	**allGIW_IAFm vs. Streamflow [lag 1 months]**	**allGIW_IAFm vs. Rain+Snowmelt [lag 0 months]**	**allGIW_IAFm vs. Rain+Snowmelt [lag 1 months]**	**allGIW_IAFm vs. Rain+Snowmelt-ET [lag 0 months]**	**allGIW_IAFm vs. Rain+Snowmelt-ET [lag 1 months]**
**CVP**	0.947	0.941	0.838	0.969	0.368	0.816
**PP**	0.567	0.000	0.057	0.542	0.232	0.361
**BAS**	0.726	0.007	0.616	0.674	0.543	0.590
**MVP**	0.909	0.282	0.553	0.397	0.867	0.612
**PL**	0.661	0.618	0.513	0.438	0.233	0.048
**CYD**	0.010	0.036	0.192	0.173	0.015	0.002
**COP**	0.033	0.008	0.013	0.002	0.122	0.222
**POC**	0.710	0.393	0.475	0.270	0.017	0.296
**DEB**	0.568	0.222	0.458	0.398	0.561	0.457
**NES**	0.160	0.010	0.014	0.529	0.521	0.257

Figure S1. Wetlandscapes and the Hydrologic Unit Code-12 (HUC12) contributing watershed to USGS stream gaging stations. Each rectangular study area has an area of 1000 km².

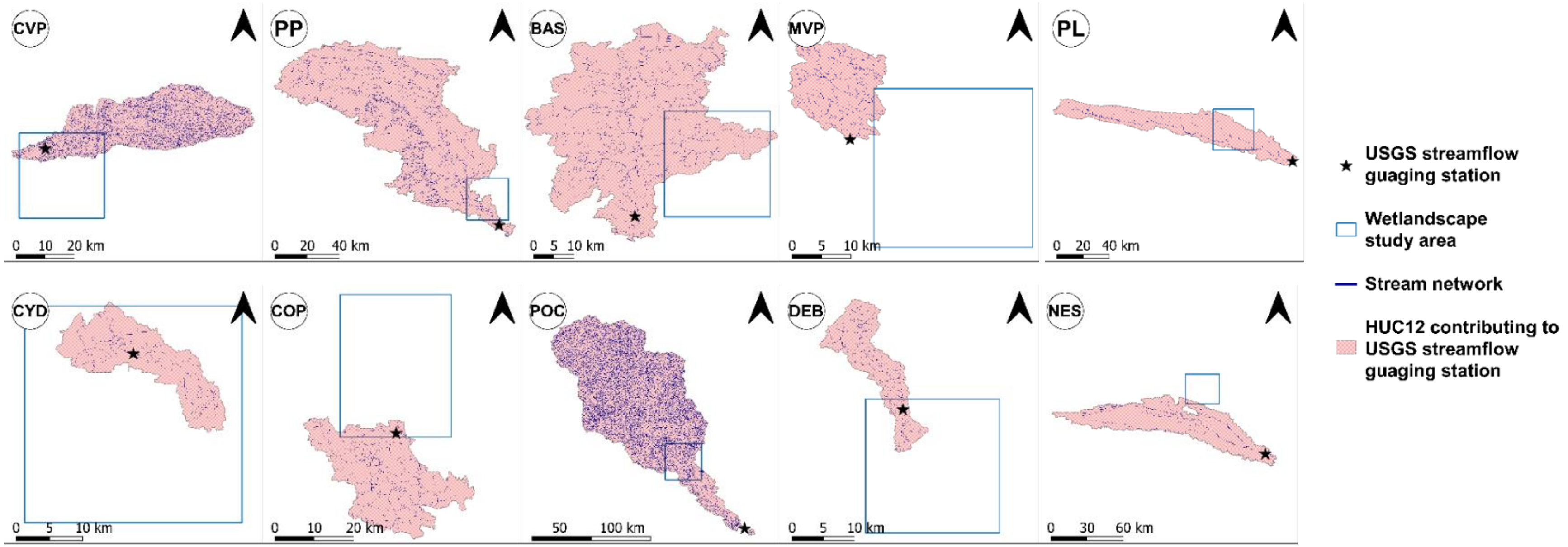

Figure S2. Heat map of the fraction of the number of years with no data within a given month (a), and the fraction of the number of GIWs for which high-quality data exists (< 5% of the area with no data) within a given month for different wetland complexes (b). The numbers on each grid indicate the magnitude of relevant variables.

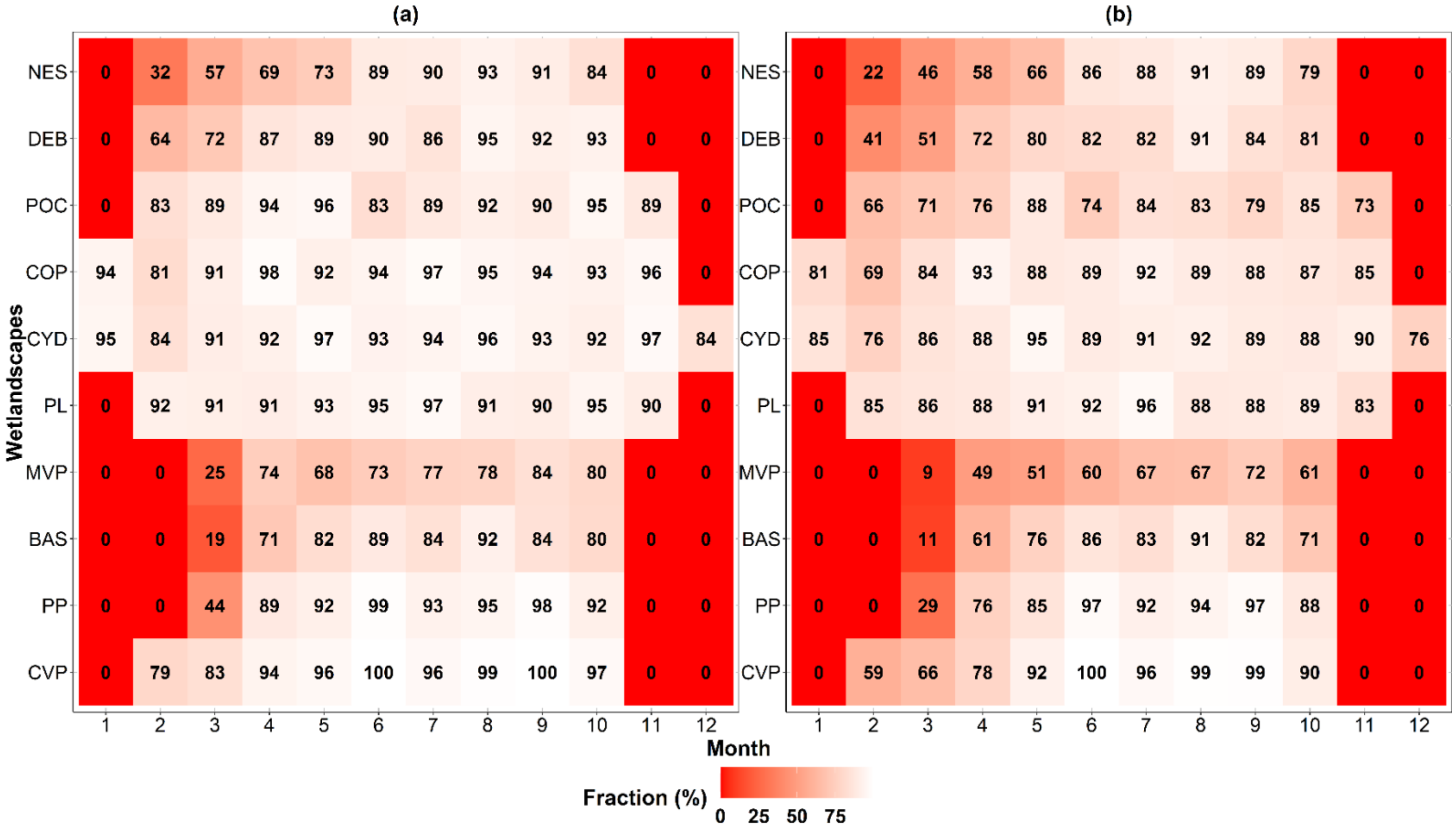

Figure S3. Scatter plot between the monthly mean of the average standard deviation of inundated area fraction over all GIWs (allGIWs_IAF$_{std}$) and monthly standard deviation of streamflow rate per unit area at the streamflow gaging stations (in m/month). The straight line is the linear regression fit. Months 1 to 12 indicate January to December.

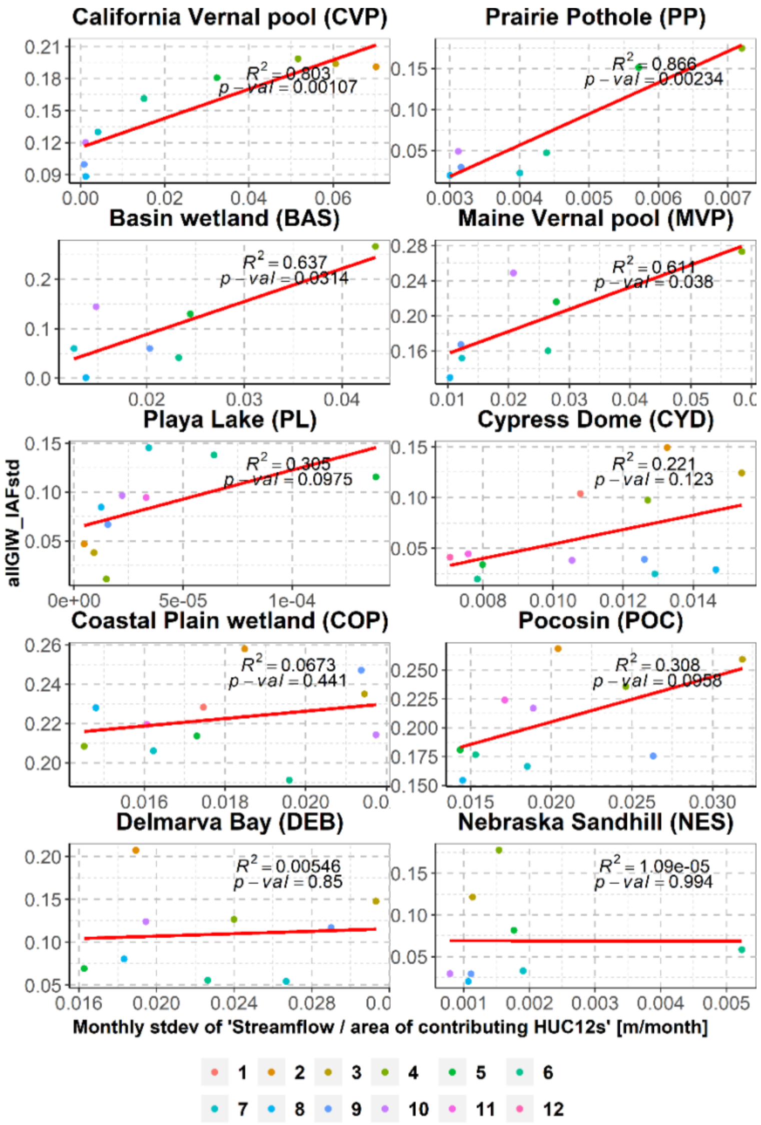

Figure S4. Scatter plot between the averaged inundation area fraction of all GIWs for each timestep (allGIW_IAF) and monthly streamflow rate per unit area of contributing Hydrologic Unit Code-12 (HUC12) (in m/month). The straight line is the linear regression fit. Months 1 to 12 indicate January to December.

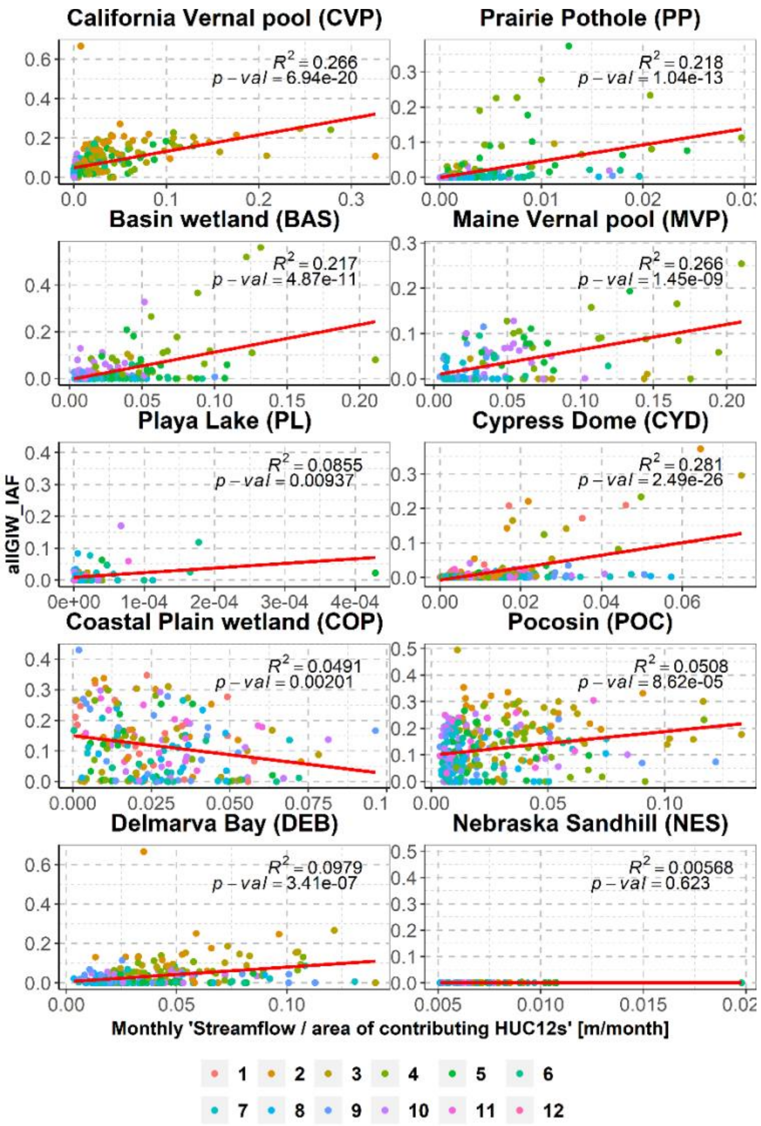

Figure S5. The average monthly fluxes for March 1984 to October 2015 in each wetland complex. Snowmelt, evapotranspiration (ET), and potential evapotranspiration (PET) estimates are from VIC NLDAS-2 data. Streamflow is obtained from waterdata.usgs.gov at gaging stations shown in Figs. 2 and S1. Data only for months used in Figure 5 are shown.

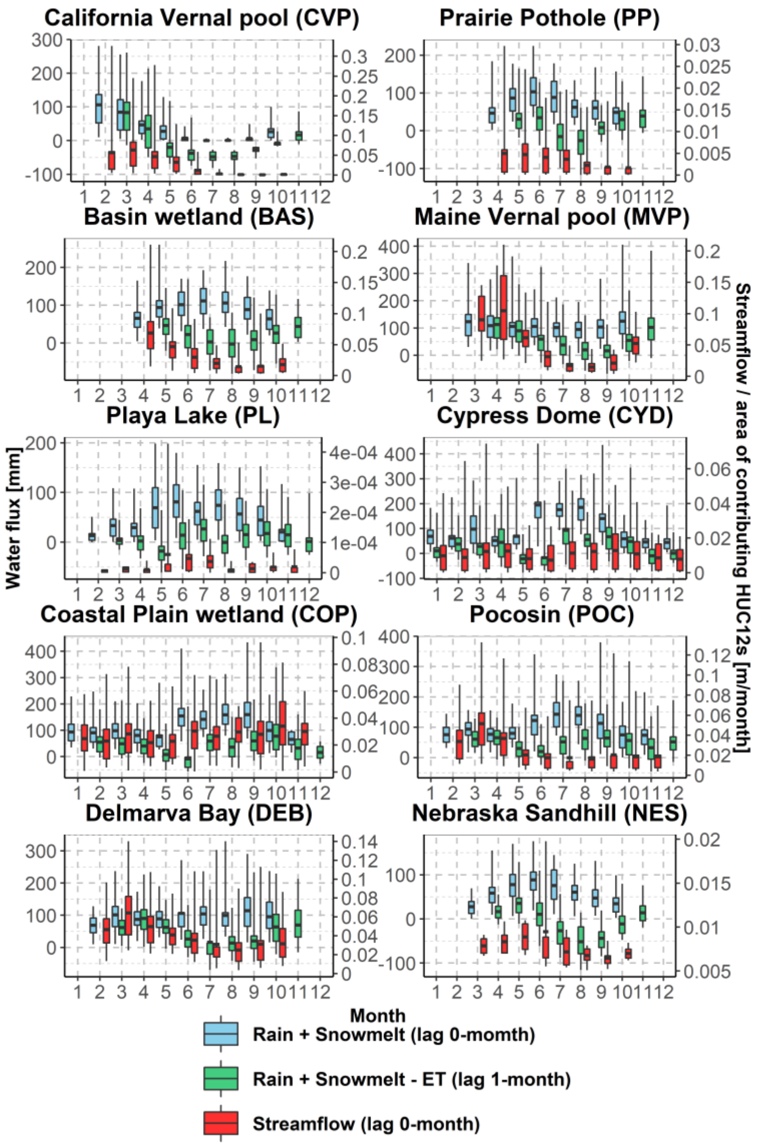

Figure S6. Conceptual figure depicting the fluxes that influence wetland inundation dynamics. ET ≡ evapotranspiration, PET ≡ potential evapotranspiration, R ≡ rainfall, and SM ≡ snowmelt.

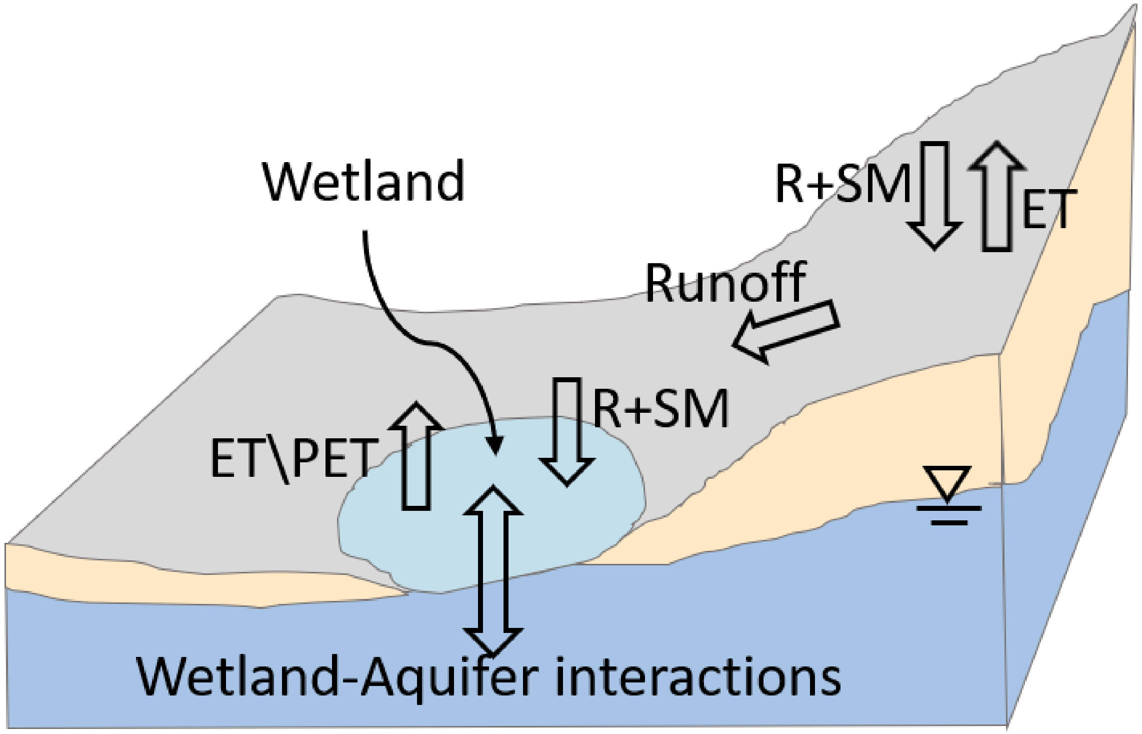

Figure S7. Area vs. perimeter relation for the wetlandscapes during the analyses period. Based on the Chow-test, p-value > 0.05 is observed for CVP, COP, POC, DEB, and NES, indicating statistically significant differences in linear regressions between the two periods.

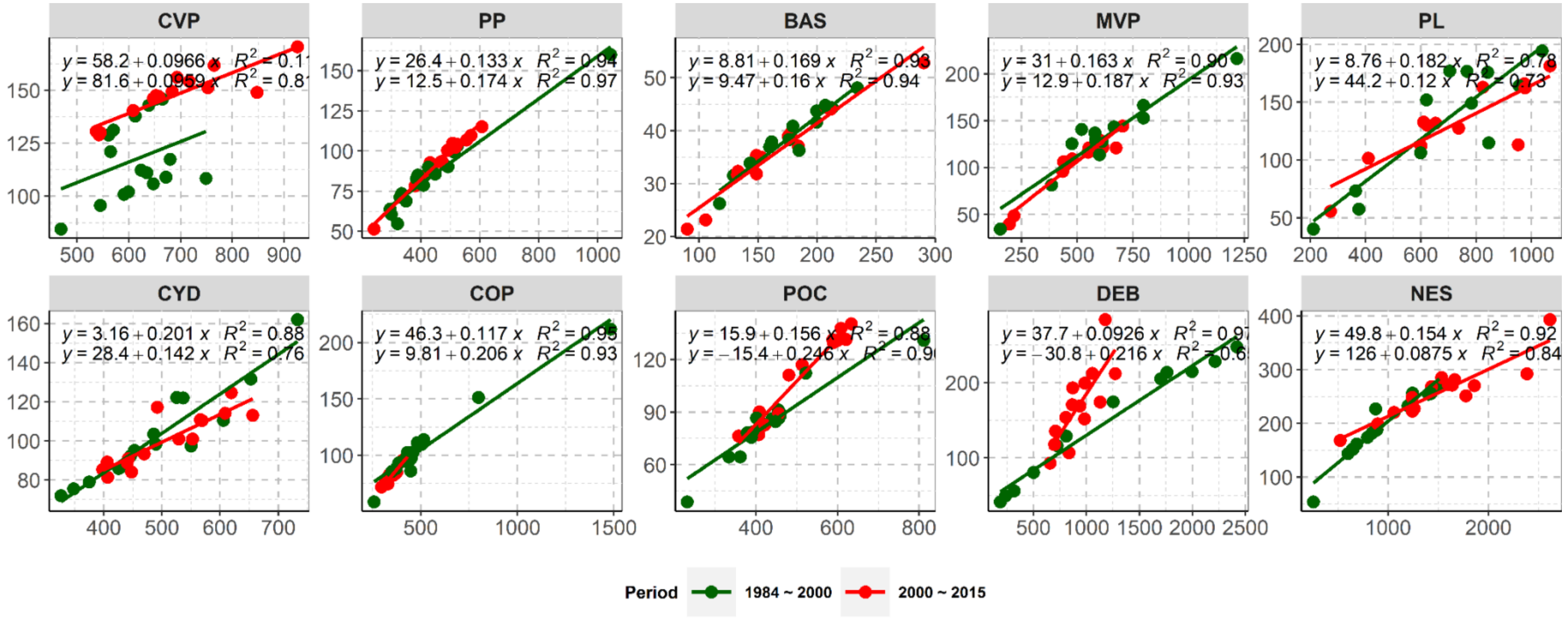

Figure S8. Observed vs. predicted seasonality of inundation, i.e., allGIW_IAF$_m$ over the two halves of the analysis period (1984-2015).

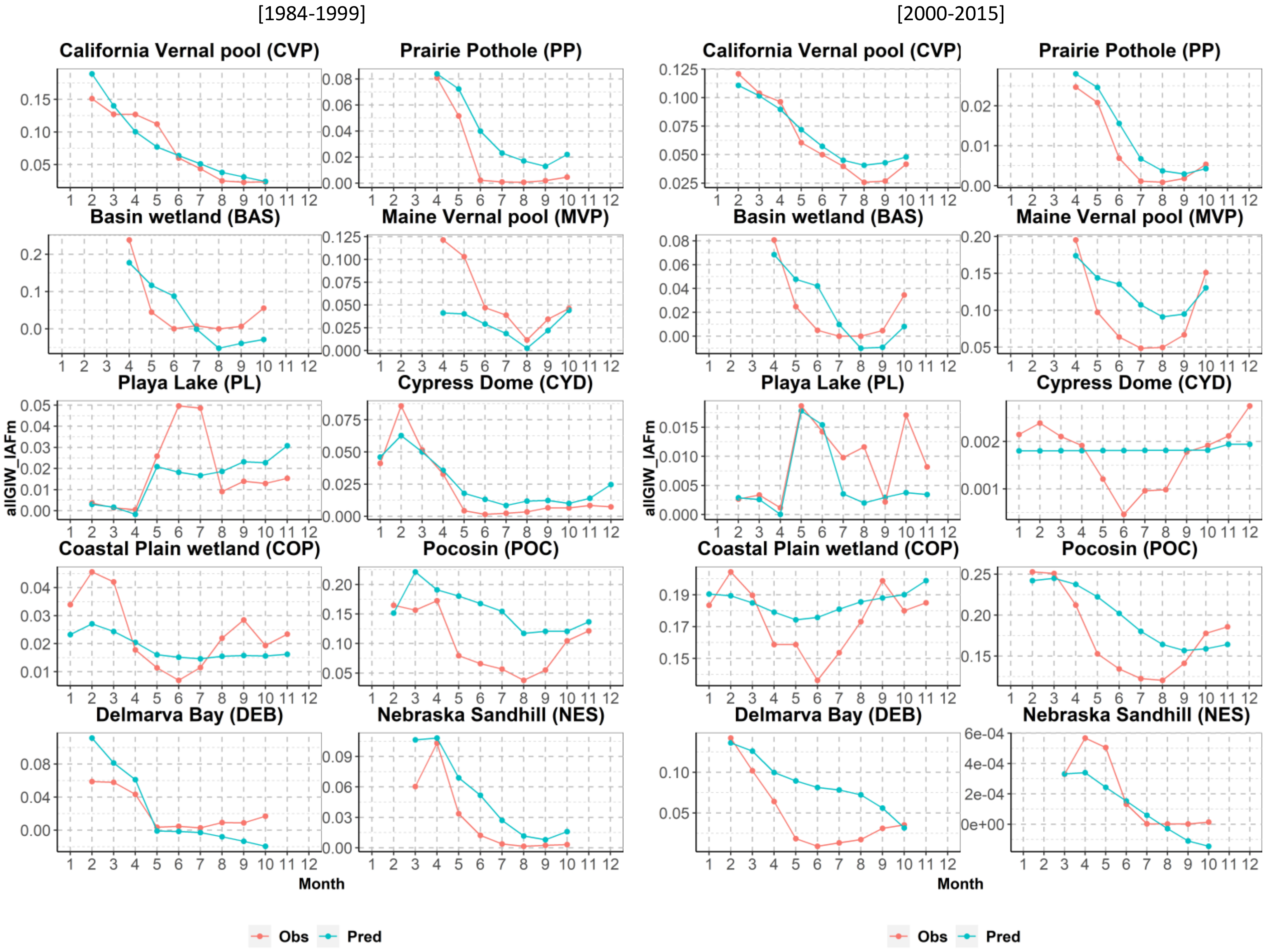

Supplement: Sup 1 [file NIHMS1805452-supplement-Sup_1.pdf]
